# Supplementary material for: Catalytic site flexibility facilitates the substrate and catalytic promiscuity of Vibrio dual lipase/transferase
Source: Nat Commun. 2023 Aug 9;14:4795. doi: 10.1038/s41467-023-40455-y (PMC10412561; doi:10.1038/s41467-023-40455-y)
Supplement: Supplementary file 1 — Supplementary information [file 41467_2023_40455_MOESM1_ESM.pdf]

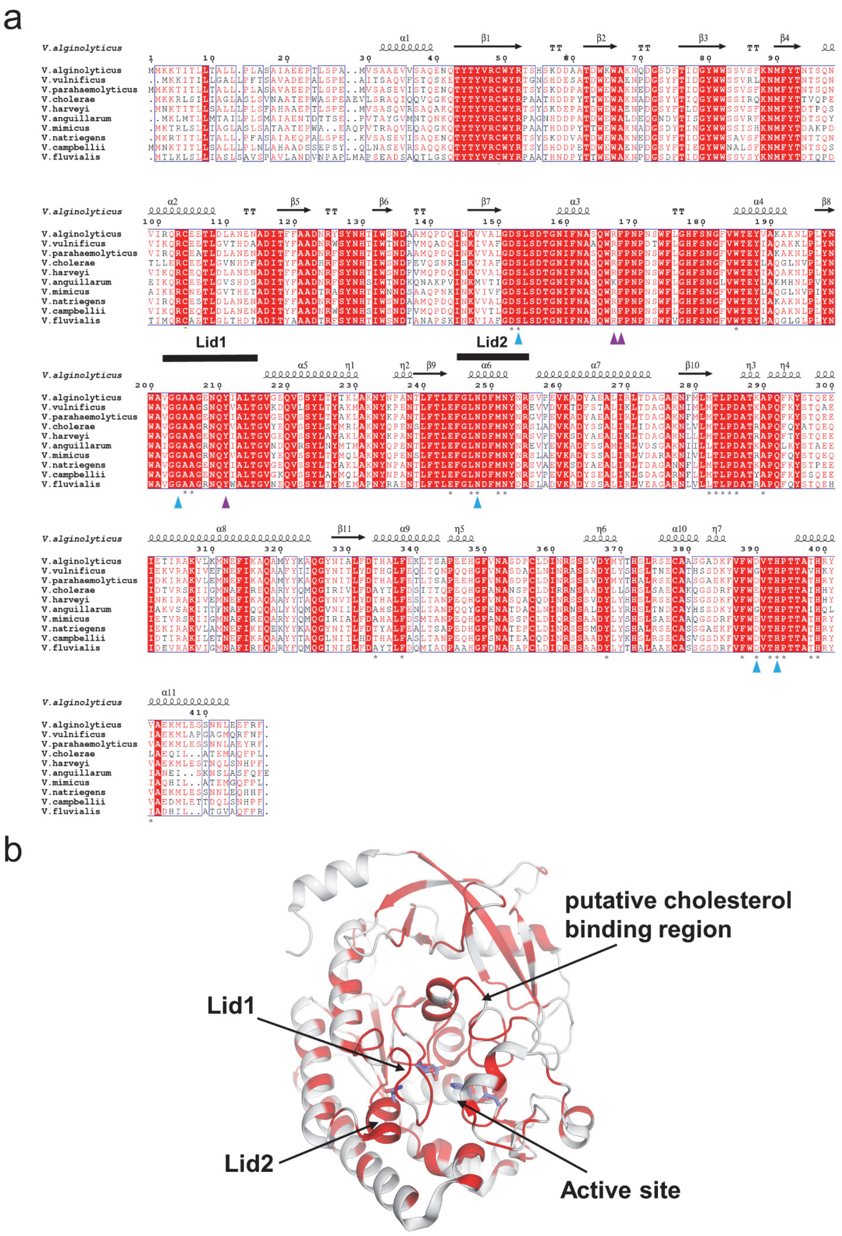

Supplementary Figure 1. Sequence and structural conservation among VDLTs. (a) Sequence alignment of VDLTs. Identical residues are shaded in red. Lid1 and Lid2 are indicated above the sequences. The catalytic residues are indicated by blue triangles. The residues constituting the acyl-binding pocket are indicated by black stars. The residues whose mutations affected cholesterol binding are indicated by purple triangles. (b) Mapping of the sequence identity of the VDLTs on the ValDLT/LAA structure. The catalytic residues are shown in stick representation.

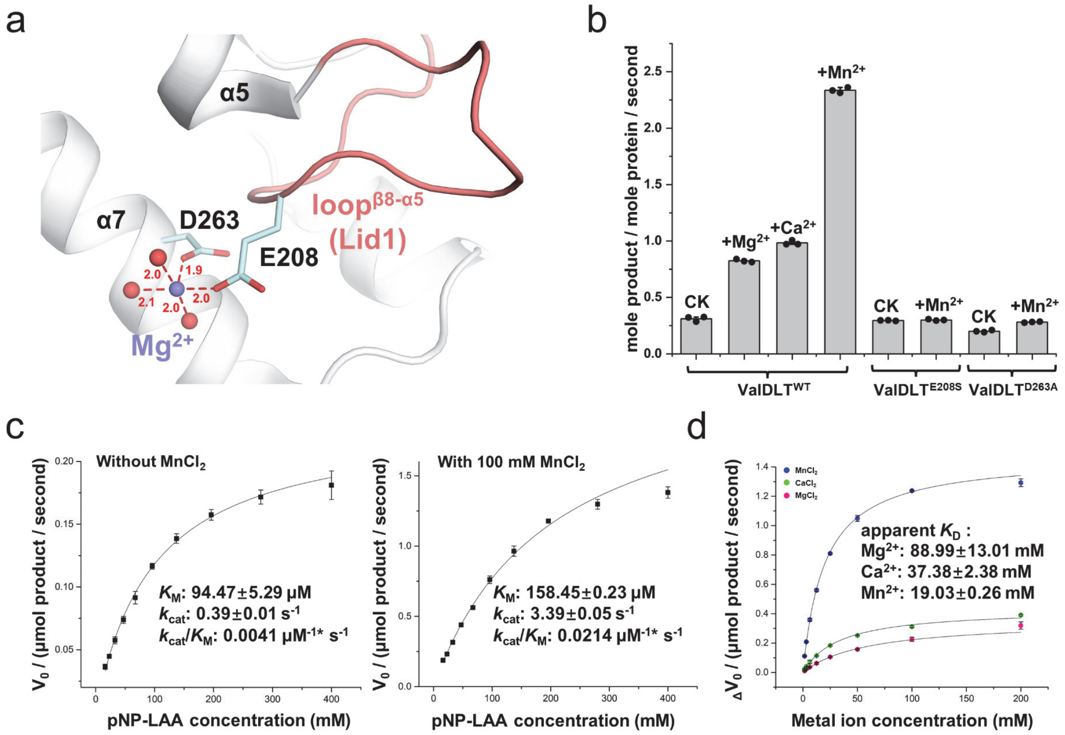

Supplementary Figure 2. Metal ions modulate Lid1 conformation and affect enzyme activity. (a) A magnesium ion (slate sphere) is coordinated by E208 of Lid1 (loop$^{β8-α5}$), D263 and water molecules (red spheres), as shown in *apo* ValDLT_B. (b) Esterase activity of ValDLT and its chelating-residue mutants toward pNP-LAA with or without divalent metal ions. (c) Kinetics data of ValDLT towards pNP-LAA in the absence and presence of 100 mM $MnCl_2$. (d) Estimation of the apparent K_D values of ValDLT for Mg^{2+}, Ca^{2+} and Mn^{2+}. The values of specific activity, apparent K_D (represented as the mean ± SD) and kinetic data (mean ± SE) were calculated from three independent measurements.

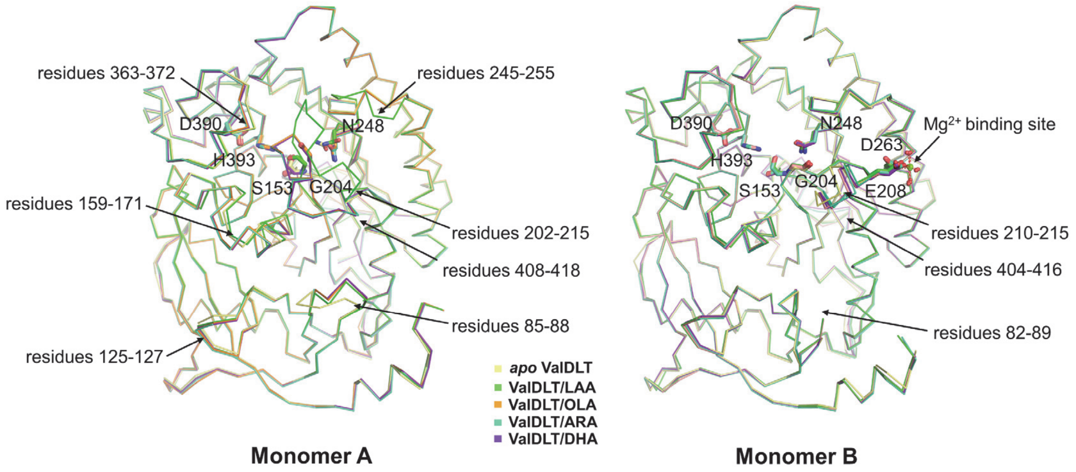

Supplementary Figure 3. Superposition of the overall structures of monomer A (left) and monomer B (right) of *apo* ValDLT, ValDLT/LAA, ValDLT/OLA, ValDLT/ARA and ValDLT/DHA. Regions with significant conformational differences are indicated. Catalytic residues are shown in stick representation. Mg^{2+} binding sites in monomer B are indicated, with the binding residues shown in stick representation.

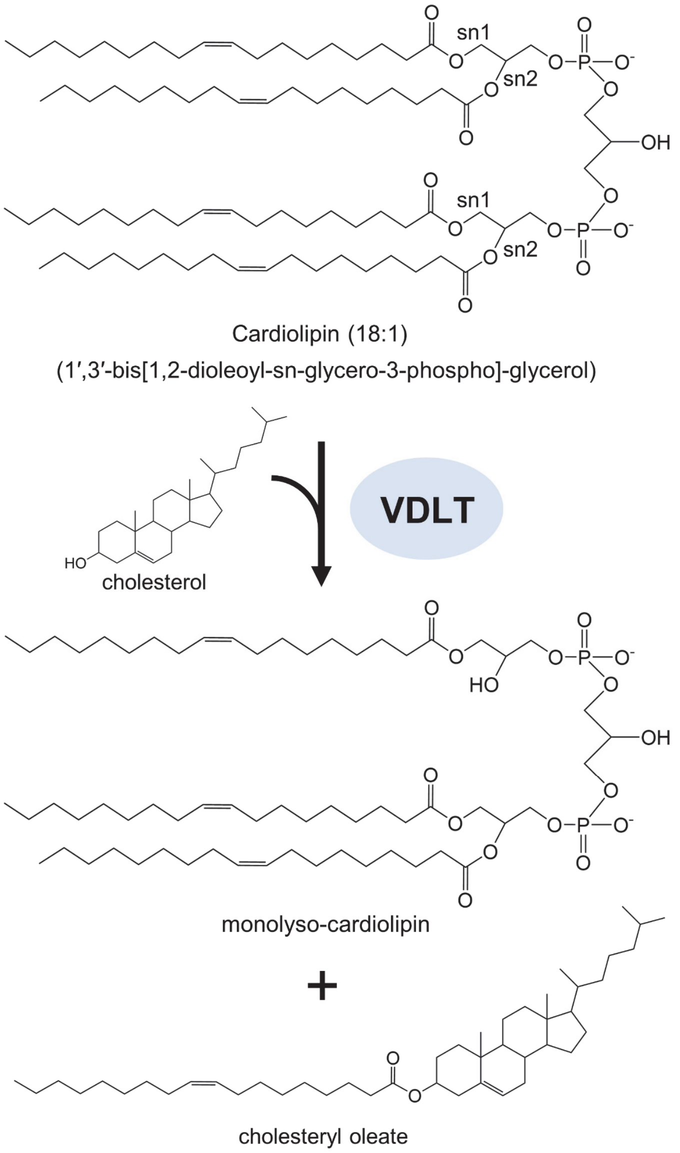

Supplementary Figure 4. Schematic description of the acyl transfer from cardiolipin (C18:1) to cholesterol, catalyzed by VDLT. For simplicity, only one acyl chain at the sn-2 position of cardiolipin (C18:1) is shown for transfer, but other acyl chains could also be transferred.

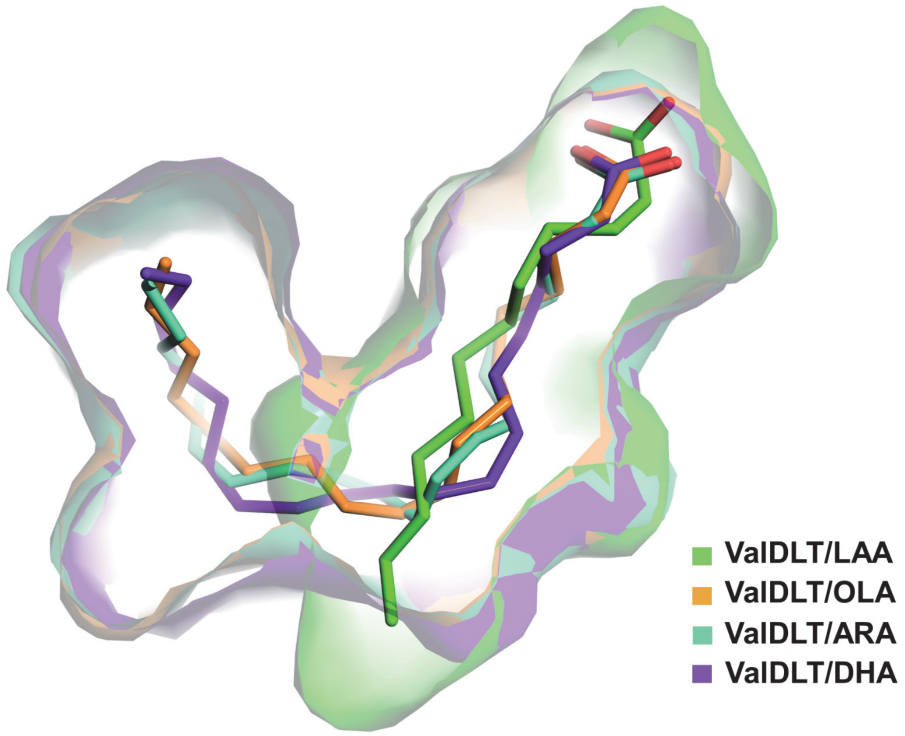

Supplementary Figure 5. Comparison of the binding modes of different fatty acids in the acyl-binding pocket of ValDLT. The ligands are shown in stick representation.

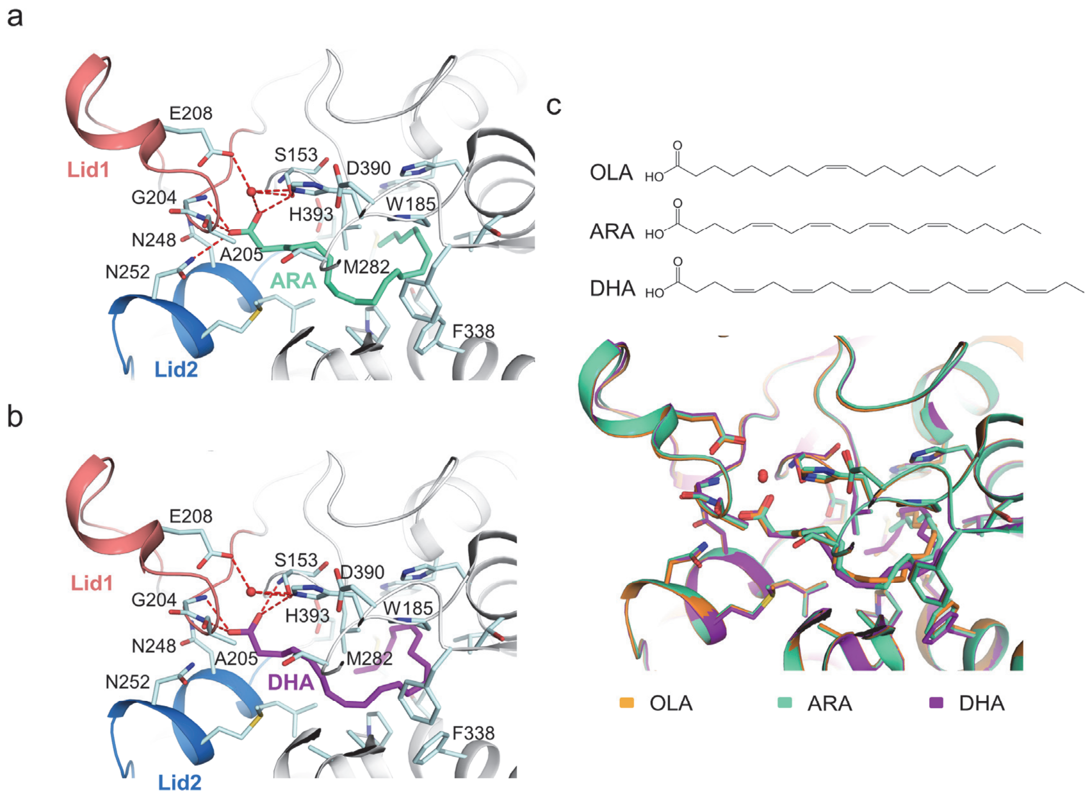

Supplementary Figure 6. The active sites of the ValDLT/ARA and ValDLT/DHA complexes. (a) ARA binding mode in the active site of the ValDLT/ARA complex. (b) DHA binding mode in the active site of the ValDLT/DHA complex. (c) Superposition of the active sites of ValDLT/OLA, ValDLT/ARA and ValDLT/DHA. The residues and ligands are shown in stick representation. Water molecules are shown as red spheres. Hydrogen bonds are represented as red dashed lines. The chemical formulas of OLA, ARA and DHA are displayed above the superposition.

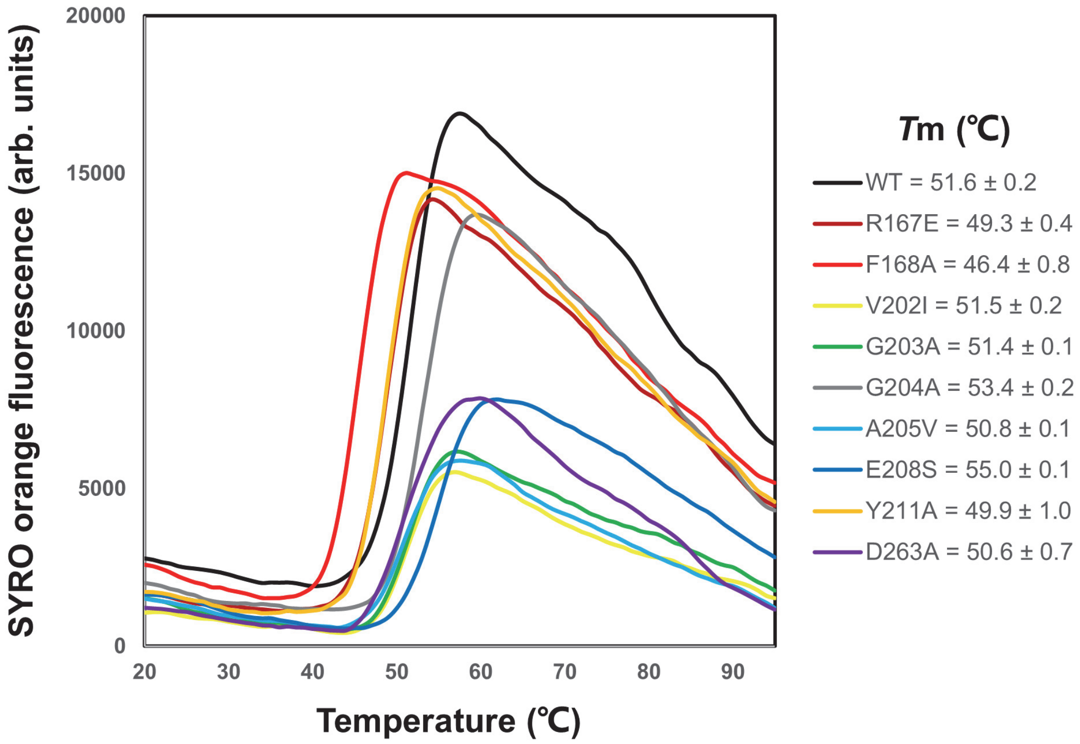

Supplementary Figure 7. *T*m values of ValDLT and its mutants estimated from the thermal shift assay. The left panel shows representative melting curves, with the fluorescence signals in arbitrary units (arb. units). The right panel lists the *T*m values (mean ± SD) calculated from three independent measurements.

Supplementary Table 1. The list of primers.

Primer Name	Sequence (5'-3')
pETM13-ValDLT-NcoI-F	agtccatgggcATGATGAAAAAAACAATCACAC
pETM13-ValDLT-his6-EcoRI-R	atgaattcttagtgatggtgatggtgatgAAAGCGAAACTCTTCTAAG
pETM13-VPA0226-NcoI-F	agtccatgggcAAAAAAACAATCACACTATTAAC
pETM13-VPA0226-his6-EcoRI-R	atgaattcttagtgatggtgatggtgatgGAAACGGTACTCTGCTAAG
pETM13-lec/VC_A0218-NcoI-F	agtccatgggcAAAAAAAGACTCTCTATTCTAATC
pETM13-lec/VC_A0218-his6-EcoRI-R	atgaattcttagtgatggtgatggtgatgAAGCGGAAATTGTGCC
ValDLT-D263A-F	TGCCGGAAGTAAAAGCGGCTTACGCTGAAGCTCTGATTC
ValDLT-D263A-R	GAATCAGAGCTTCAGCGTAAGCCGCTTTTACTTCCGGCA
ValDLT-E208S-F	GTTGGTGGCGCAGCAGGTTCAAACCAATACATCGCGCTA
ValDLT-E208S-R	TAGCGCGATGTATTGGTTTGAACCTGCTGCGCCACCAAC
ValDLT-H393A-F	GTGTTCTGGGATGTGACTGCCCCAACCACAGCAACGCA
ValDLT-H393A-R	TGCGTTGCTGTGGTTGGGGCAGTCACATCCCAGAACAC
ValDLT-V202I-F	CCGCTATACAACTGGGCAATTGGTGGCGCAGCAGGTGAA
ValDLT-V202I-R	TTCACCTGCTGCGCCACCAATTGCCCAGTTGTATAGCGG
ValDLT-G203A-F	CTATACAACTGGGCAGTTGCTGGCGCAGCAGGTGAAAAC
ValDLT-G203A-R	GTTTTCACCTGCTGCGCCAGCAACTGCCCAGTTGTATAG
ValDLT-A205V-F	AACTGGGCAGTTGGTGGCGTAGCAGGTGAAAACCAATAC
ValDLT-A205V-R	GTATTGGTTTTCACCTGCTACGCCACCAACTGCCCAGTT
ValDLT-G204A-F	TACAACTGGGCAGTTGGTGCCGCAGCAGGTGAAAACCAA
ValDLT-G204A-R	TTGGTTTTCACCTGCTGCGGCACCAACTGCCCAGTTGTA
ValDLT-R167E-F	CTTTAACGCGTCCCAATGGGAGTTCCCTAACCCGAATAGC
ValDLT-R167E-R	GCTATTCGGGTTAGGGAACTCCCATTGGGACGCGTTAAAG
ValDLT-F168A-F	ACGCGTCCCAATGGCGCGCCCCTAACCCGAATAGCTG
ValDLT-F168A-R	CAGCTATTCGGGTTAGGGGCGCGCCATTGGGACGCGT
ValDLT-Y211A-F	CGCAGCAGGTGAAAACCAAGCCATCGCGCTAACTGGTGT
ValDLT-Y211A-R	ACACCAGTTAGCGCGATGGCTTGGTTTTCACCTGCTGCG

Supplementary Table 2. Crystallographic statistics.

Parameter	*ValDLT*	*ValDLT/LAA*	*ValDLT/OLA*	*ValDLT/ARA*	*ValDLT/DHA*
Data collection statistics					
**X-ray source**	*BL19U, SSRF*	*BL18U, SSRF*	*BL19U, SSRF*	*BL19U, SSRF*	*BL19U, SSRF*
**Wavelength (Å)**	0.9788	0.9792	0.9785	0.9785	0.9785
**Space group**	$P2_1$	$P2_1$	$P2_1$	$P2_1$	$P2_1$
**Unit-cell dimensions**<br>**(Å or °)**	*a* = 65.93<br>*b* = 71.24<br>*c* = 83.65<br>*β* = 101.3	*a* = 65.41<br>*b* = 72.62<br>*c* = 82.99<br>*β* = 102.4	*a* = 65.81<br>*b* = 71.87<br>*c* = 83.10<br>*β* = 101.5	*a* = 66.10<br>*b* = 72.89<br>*c* = 83.90<br>*β* = 101.6	*a* = 65.67<br>*b* = 72.13<br>*c* = 83.49<br>*β* = 101.6
**Resolution (Å)**	53.78−1.809<br>(1.840−1.809)	63.89−1.940<br>(1.974−1.940)	56.30−1.931<br>(1.964−1.931)	64.75−1.980<br>(2.014−1.980)	81.78−2.008<br>(2.014−2.008)
**Unique reflections**	68600 (3412)	55927 (2776)	57066 (2812)	54484 (2731)	51148 (479)
**Completeness (%)**	98.8 (98.1)	99.3 (98.4)	99.9 (100)	99.9 (99.9)	99.9 (100)
**Mean I/sigma (I)**	12.9 (2.3)	18.2 (2.3)	20.2 (2.5)	13.8 (2.1)	14.8 (2.0)
**Multiplicity**	6.9 (6.9)	6.8 (6.9)	6.7 (6.8)	6.5 (6.6)	6.7 (6.3)
R_{merge} **(%)**	8.0 (70.9)	6.5 (70.2)	4.7 (59.6)	8.7 (91.9)	7.6 (85.0)
$R_{measure}$ **(%)**	8.7 (76.7)	7.1 (75.9)	5.1 (64.5)	9.4 (99.8)	8.3 (92.7)
R_{pim} **(%)**	3.3 (28.8)	2.7 (28.7)	2.0 (24.4)	3.7 (38.5)	3.2 (36.5)
$CC_{1/2}$ **(%)**	99.8 (86.3)	99.9 (92.1)	99.9 (91.5)	99.8 (76.7)	99.7 (86.5)
Refinement statistics					
**Resolution range (Å)**	53.78−1.81	28.40−1.94	56.30−1.93	64.75−1.98	81.78−2.01
R_{work} / R_{free} **(%)** [a]	18.0/20.4	19.3/23.1	19.5/22.1	19.3/22.7	19.1/22.5
**Modeled residues**	Chain A:<br>30−203,<br>215−416<br>Chain B:<br>31−416	Chain A:<br>28−416<br>Chain B:<br>30−83,<br>88−416	Chain A:<br>28−416<br>Chain B:<br>31−83,<br>88−416	Chain A:<br>29−84,<br>88−418<br>Chain B:<br>30−416	Chain A:<br>29−418<br>Chain B:<br>30−416
**Ligands**	4 NDSB-201<br>5 HO-PEG5-OH<br>5 HO-PEG2-OH<br>2 Mg^{2+}	1 LAA<br>3 HO-PEG2-OH<br>1 Mg^{2+}	1 OLA<br>1 HO-PEG5-OH<br>1 Mg^{2+}	1 ARA<br>2 NDSB-201<br>2 Mg^{2+}	1 DHA<br>2 NDSB-201<br>2 Mg^{2+}
**Water molecules**	307	274	201	313	154
**Bond lengths (Å)**	0.010	0.010	0.010	0.010	0.010
**Bond angles (°)**	0.93	0.97	0.97	0.97	1.00
**Ramachandran outliers (%)**	0.1	0.5	0.1	0.1	0.1
PDB code	**8H09**	**8H0A**	**8H0B**	**8H0C**	**8H0D**

Values in parentheses represent the highest resolution shell.

[a] ~5% of the reflections were selected randomly for calculating R_{free}.
