# Supplementary material for: Catalytic site flexibility facilitates the substrate and catalytic promiscuity of Vibrio dual lipase/transferase
Source: Nat Commun. 2023 Aug 9;14:4795. doi: 10.1038/s41467-023-40455-y (PMC10412561; doi:10.1038/s41467-023-40455-y)
Supplement: Supplementary file 3 — Reporting Summary [file 41467_2023_40455_MOESM3_ESM.pdf]

nature portfolio

Corresponding author(s): Qingjun Ma

Last updated by author(s): Jul 14, 2023

Reporting Summary

Nature Portfolio wishes to improve the reproducibility of the work that we publish. This form provides structure for consistency and transparency in reporting. For further information on Nature Portfolio policies, see our Editorial Policies and the Editorial Policy Checklist.

Statistics

For all statistical analyses, confirm that the following items are present in the figure legend, table legend, main text, or Methods section.

n/a	Confirmed	
☐	☒	The exact sample size (*n*) for each experimental group/condition, given as a discrete number and unit of measurement
☐	☒	A statement on whether measurements were taken from distinct samples or whether the same sample was measured repeatedly
☒	☐	The statistical test(s) used AND whether they are one- or two-sided<br>*Only common tests should be described solely by name; describe more complex techniques in the Methods section.*
☒	☐	A description of all covariates tested
☒	☐	A description of any assumptions or corrections, such as tests of normality and adjustment for multiple comparisons
☐	☒	A full description of the statistical parameters including central tendency (e.g. means) or other basic estimates (e.g. regression coefficient) AND variation (e.g. standard deviation) or associated estimates of uncertainty (e.g. confidence intervals)
☒	☐	For null hypothesis testing, the test statistic (e.g. *F*, *t*, *r*) with confidence intervals, effect sizes, degrees of freedom and *P* value noted<br>*Give P values as exact values whenever suitable.*
☒	☐	For Bayesian analysis, information on the choice of priors and Markov chain Monte Carlo settings
☒	☐	For hierarchical and complex designs, identification of the appropriate level for tests and full reporting of outcomes
☒	☐	Estimates of effect sizes (e.g. Cohen's *d*, Pearson's *r*), indicating how they were calculated

Our web collection on statistics for biologists contains articles on many of the points above.

Software and code

Policy information about availability of computer code

Data collection	The X-ray diffraction data were collected with the in-site software Blu-Ice at the beamlines BL18U1 and BL19U1 of Shanghai Synchrotron Radiation Facility (SSRF).
Data analysis	autoPROC (1.04 <2015-03-16 01:21:00>, 1.05 <2017-12-19 01:53:57>), XDS (MAR 15, 2019, BUILT ON 20190315; JAN 31, 2020, BUILT ON 20200417; JAN 10, 2022, BUILT ON 20220220), AIMLESS 0.5.29, autoBUSTER 2.10.2, CCP4-7.0, PHASER 2.7.17, Coot 0.8.7 EL, GRADE server, MolProbity server, Pymol 2.5.2, Origin 2018

For manuscripts utilizing custom algorithms or software that are central to the research but not yet described in published literature, software must be made available to editors and reviewers. We strongly encourage code deposition in a community repository (e.g. GitHub). See the Nature Portfolio guidelines for submitting code & software for further information.

Data

Policy information about availability of data

All manuscripts must include a data availability statement. This statement should provide the following information, where applicable:

- Accession codes, unique identifiers, or web links for publicly available datasets
- A description of any restrictions on data availability
- For clinical datasets or third party data, please ensure that the statement adheres to our policy

The coordinates and diffraction data generated in this study have been deposited in the PDB (www.rcsb.org) with accession numbers of 8H09 (apo ValDLT), 8H0A (ValDLT/LAA), 8H0B (ValDLT/OLA), 8H0C (ValDLT/ARA) and 8H0D (ValDLT/DHA). The crystal structure of VvPlpA used in this study is available in the PDB under the

accession code 6JKZ. All other data are contained within the manuscript and the supplementary information file. Source data are provided with this paper.

Human research participants

Policy information about studies involving human research participants and Sex and Gender in Research.

Reporting on sex and gender	This issue is not relevant to this study.
Population characteristics	This issue is not relevant to this study.
Recruitment	This issue is not relevant to this study.
Ethics oversight	This issue is not relevant to this study.

Note that full information on the approval of the study protocol must also be provided in the manuscript.

Field-specific reporting

Please select the one below that is the best fit for your research. If you are not sure, read the appropriate sections before making your selection.

☒ Life sciences ☐ Behavioural & social sciences ☐ Ecological, evolutionary & environmental sciences

For a reference copy of the document with all sections, see nature.com/documents/nr-reporting-summary-flat.pdf

Life sciences study design

All studies must disclose on these points even when the disclosure is negative.

Sample size	The sample sizes for the biochemical assays were equal to or greater than 3 following the traditional rule, which is sufficient to generate error bars to evaluate the quality of the measurements.
Data exclusions	The X-ray data were processed by standard software and no data were excluded illegally. No data were excluded in the biochemical assays.
Replication	All biochemical experiments were independently repeated at least 3 times. All attempts at replication were successful.
Randomization	In the biochemical assays samples were not allocated to groups and thus no randomization was needed. About 5% of the diffraction data were randomly selected by the software autoPROC in a default manner to calculate Rfree.
Blinding	No blinding protocols were used, because no clinical trials were conducted.

Reporting for specific materials, systems and methods

We require information from authors about some types of materials, experimental systems and methods used in many studies. Here, indicate whether each material, system or method listed is relevant to your study. If you are not sure if a list item applies to your research, read the appropriate section before selecting a response.

Materials & experimental systems

n/a	Involved in the study	
☒	☐	Antibodies
☒	☐	Eukaryotic cell lines
☒	☐	Palaeontology and archaeology
☒	☐	Animals and other organisms
☒	☐	Clinical data
☒	☐	Dual use research of concern

Methods

n/a	Involved in the study	
☒	☐	ChIP-seq
☒	☐	Flow cytometry
☒	☐	MRI-based neuroimaging
